# The expression of ferroptosis-related proteins GPX4 and PTGS2 in colonic tissues of pediatric Crohn’s disease

**DOI:** 10.1016/j.jped.2025.101431

**Published:** 2025-08-18

**Authors:** Shuanghong Zhang, Chunzhi Kang, Xiaoxiao He, Hui Huang, Qian Xiao

**Affiliations:** a Department of Digestive Medicine, Jiangxi Province Children's Hospital, The Affiliated Children's Hospital of Nanchang Medical College, Nanchang, China; b Department of Pathology, Jiangxi Province Children's Hospital, The Affiliated Children's Hospital of Nanchang Medical College, Nanchang, China

**Keywords:** Pediatric, Crohn's disease, Ferroptosis, Immunohistochemistry

## Abstract

**Objective:**

To investigate the expression of ferroptosis-related proteins GPX4 and PTGS2 in pediatric Crohn’s disease (CD).

**Methods:**

Pediatric CD colonic tissues by colonoscopy biopsy were selected as the subjects. The Pediatric Crohn’s Disease Activity Index (PCDAI) was used to assess the disease activity of CD. Immunohistochemistry was used to detect the expression of GPX4 and PTGS2.

**Results:**

30 CD cases were collected, with an average age of 12.36±2.65 years, including 9 in the mild activity phase, 8 in the moderate activity phase, and 13 in the severe activity phase. The positive expression rate of GPX4 in CD colonic tissues was lower than that in normal colonic tissues (*p* = 0.02); PTGS2 in CD colonic tissues was higher than that in normal colonic tissues (*p* = 0.004). Based on the average optical density (AOD) values of the positive reactants, the relative expression levels of GPX4 in CD colonic tissues were lower than those in normal colonic tissues (*p* = 0.02); PTGS2 in CD colonic tissues were higher than those in normal colonic tissues (*p* = 0.000). The staining scores of GPX4 decreased with increasing CD disease activity (*p* < 0.001); PTGS2 increased with increasing CD disease activity (*p* < 0.001). GPX4 was negatively correlated with CD disease activity (*r* = −0.326); PTGS2 was positively correlated with CD disease activity (*r* = 0.299).

**Conclusion:**

GPX4 and PTGS2 may be involved in the process of ferroptosis in CD intestinal epithelial cells and are correlated with the severity of pediatric CD.

## Introduction

Crohn’s disease (CD) is a subtype of inflammatory bowel disease (IBD), characterized by chronic, non-specific intestinal inflammation of unknown etiology. It can affect various layers of the gastrointestinal tract, from the mucosa to the serosa, as well as the surrounding mesentery. In recent years, the incidence of pediatric CD has been rising rapidly [1,2]. Compared to adults, pediatric CD involves more extensive intestinal inflammation and progresses more quickly. Although the exact pathogenesis of CD remains unclear, it is believed to result from a combination of factors, including genetic susceptibility, environmental influences, infections, the gut microbiota, and immune responses [3].

Ferroptosis is a newly identified form of regulated cell death that is iron-dependent, characterized by the accumulation of iron-dependent reactive oxygen species (ROS) and lipid peroxidation (LPO) [4]. Ferroptosis is induced via multiple pathways, such as iron overload, depletion of glutathione (GSH), inactivation of glutathione peroxidase 4 (GPX4), and the accumulation of ROS and LPO, which together mediate damage to the colonic mucosal barrier in CD [5]. During ferroptosis, GPX4 and prostaglandin-endoperoxide synthase (PTGS2) are considered biomarkers of ferroptosis, involved in the enzyme-catalyzed lipid peroxidation process [6].

This is an exploratory study aimed at investigating the expression of ferroptosis-related proteins GPX4 and PTGS2 in pediatric CD. This study used immunohistochemical methods to detect the expression of GPX4 and PTGS2 in colonic tissues of pediatric CD, analyzing the correlation between their expression and the disease activity of CD, and providing a new perspective for further research into the role of GPX4 and PTGS2 in the mechanism of CD.

## Materials

### Study subjects

A total of 30 pediatric CD cases first diagnosed at Jiangxi Province Children's Hospital between March 2021 and August 2023 were selected, along with 30 cases of normal colonic tissue, age- and sex-matched, from colonic tissue by colonoscopy biopsy during the same period, as the control group. The diagnostic criteria for CD followed the 2014 Porto Consensus guidelines of ECCO/ESPGHAN on the medical management of pediatric Crohn's disease, as well as the 2019 Chinese expert consensus on the diagnosis and management of pediatric inflammatory bowel disease [7,8]. Inclusion criteria: (1) meeting the diagnostic criteria for CD; (2) age < 16 years; (3) complete clinical data and well-preserved colonic tissue by colonoscopy biopsy. Exclusion criteria: (1) inability to diagnose CD; (2) presence of other chronic infectious intestinal diseases or intestinal malignancies; (3) incomplete clinical data. This study was reviewed and approved by the Jiangxi Province Children's Hospital ethics committee (JXSETYY-YXKY–20240112), with a waiver of informed consent for retrospective studies.

### PCDAI scoring and grouping

Patient data, including age, gender, disease duration, height, weight, laboratory tests, and colonoscopy findings, were collected from the hospital's electronic medical records. The Pediatric Crohn’s Disease Activity Index (PCDAI) was used to assess the disease activity of CD, referring to the "Evaluation of children with inflammatory bowel diseases" [9] . PCDAI scores were based on factors such as abdominal pain, stool characteristics, general condition, body weight, height, abdominal signs, perianal disease, extraintestinal manifestations, hematocrit, erythrocyte sedimentation rate, and albumin. A PCDAI score < 10 was defined as clinical remission, 10.0 ∼ 27.5 as mild activity, 30.0 ∼ 37.5 as moderate activity, and 40.0 ∼ 100.0 as severe activity [10].

### Experimental methods

#### Specimen collection

Colonic tissue by colonoscopy biopsy from CD cases first diagnosed at the pathology department of Jiangxi Province Children's Hospital were collected, along with colonic biopsy tissues from age- and sex-matched cases of normal colonic tissue from the same period. All colonic specimens used in this study were the remaining wax block tissues after clinical pathological examination.

#### Immunohistochemical methods

Appropriate colonic tissue sections by colonoscopy biopsy were selected, and conventional dewaxing and dehydration were performed using xylene and absolute ethanol. Antigen retrieval was carried out using an antigen retrieval solution, and endogenous peroxidase was blocked. Primary antibodies were added and incubated at 4 °C overnight. After three PBS washes, secondary antibodies were added and incubated at room temperature for 1 hour. After another three PBS washes, DAB solution was applied for color development, followed by hematoxylin counterstaining, dehydration, and neutral resin sealing. Microscopic images were taken afterward.

### Interpretation of immunohistochemical staining results

#### Qualitative interpretation

A semi-quantitative method was used to record the expression intensity of GPX4 and PTGS2 in colonic tissues of different groups. Brownish-yellow or brown precipitates in the cells under a light microscope were considered positive expression, and the density of the precipitates represented the amount of antigen. Cells without brownish-yellow or brown precipitates were considered negative for expression. Staining results were classified based on the percentage of positive cells as follows: negative (–): < 5 %; weak positive (±): 5 % ∼ 24 %; positive (+): 25 % ∼ 49 %; and strong positive (++): > 50 %.

#### Quantitative interpretation

According to the standards proposed by Prasad et al., [11] the average optical density (AOD) was calculated using Image-Pro Plus software to analyze the relative expression levels. The immunoreactivity score (IRS) was equal to the product of staining intensity (SI) and the percentage of positive cells (PP), i.e., IRS = SI × PP. Positive expression was indicated by brownish-yellow or brown granules in the cells. Under a 400 × microscope with the same light source, brightness, and contrast conditions, five fields were randomly selected from images at the same magnification. Image-Pro Plus software was used to analyze the relative expression levels, calculating the average AOD value, which represented the percentage of positively stained areas.

### Statistical analysis

Data were organized and statistically analyzed using SPSS 26.0 and GraphPad Prism software. Categorical data were expressed as frequencies and percentages ( %), and comparisons between groups were made using the χ² test and Fisher test. Continuous data were expressed as mean ± standard deviation ($\bar{\chi }$ ± *s*), and comparisons between multiple groups were made using Independent Samples *t*-test. Correlation analyses were performed using a Pearman analysis, with *p* < 0.05 considered statistically significant.

## Results

### General conditions and disease activity

A total of 30 pediatric CD cases were collected, including 22 males and 8 females, with an average age of 12.36 ± 2.65 years. Normal colon tissues were obtained from 30 cases, including 18 males and 12 females, with an average age of 9.36 ± 2.71 years. According to the PCDAI, the classification of disease activity levels for pediatric CD is shown in Table 1. There were no statistically significant differences in gender composition or age between cases of CD colonic tissue and normal colonic tissue (*p* > 0. 05).

**Table 1:** Basic information of the cases and the PCDAI subgroup.

Group		CD (*n* = 30)	Normal (*n* = 30)
Gender [n( %)]	Male	22 (73.33)	18 (60)
	Female	8 (26.67)	12 (40)
Age ($\bar{\chi }$ ± *s* years)		12.36 ± 2.65	9.36 ± 2.71
PCDAI Subgroup [n( %)]	Remission phase	0	−
	Mild activity phase	9 (30.00)	−
	Moderate activity phase	8 (26.77)	−
	Severe activity phase	13 (43.33)	−

### Expression of GPX4 and PTGS2 in CD and normal colonic tissues

**Figure 1:**
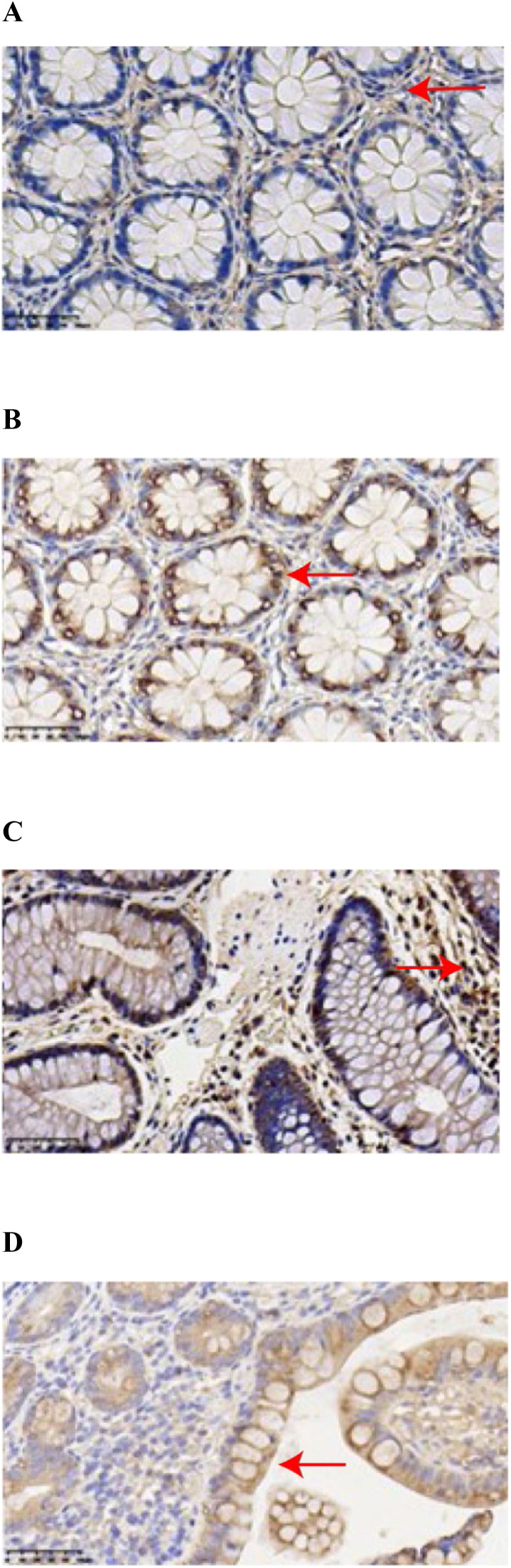
Immunohistochemical Staining Results of GPX4 and PTGS2 in CD and Normal Colonic Tissues. **(**A) GPX4 showed weak positive expression in CD colonic tissues, characterized by light color and few granules. (B) PTGS2 showed strong positive expression in CD colonic tissues, characterized by many brownish-yellow or brown granules. (C) GPX4 showed strong positive expression in normal colonic tissues, characterized by many brownish-yellow or brown granules. (D) PTGS2 showed weak positive expression in normal colonic tissues, characterized by light color and few granules.

The expressions of GPX4 and PTGS2 in the cells were indicated by the presence of brownish-yellow or brown granules, with positive levels ranging from “± to ++.” GPX4 showed weak positive expression in CD colonic tissues, characterized by light color and few granules, with positive levels ranging from “± to +” (Figure 1A); PTGS2 showed strong positive expression in CD colonic tissues, characterized by many brownish-yellow or brown granules, with positive levels ranging from “+ to ++” (Figure 1B). In normal colonic tissues, GPX4 showed strong positive expression, characterized by many brownish-yellow or brown granules, with positive levels ranging from “+ to ++” (Figure 1C); PTGS2 showed weak positive expression, characterized by light color and few granules, with positive levels ranging from “± to +” (Figure 1D). The staining scores of GPX4 and PTGS2 in CD colonic tissues and normal colonic tissues are shown in Table 2. The positive expression rate of GPX4 in CD colonic tissues was lower than in normal colonic tissues (*p* = 0.02), while the positive expression rate of PTGS2 in CD colonic tissues was higher than in normal colonic tissues (*p* = 0.004).

**Table 2:** The expressions of GPX4 and PTGS2 in the CD and normal colonic tissues.

Group	Case number	GPX4				PTGS2			
		Positive	Negative	Positive rate ( %)	*P*	Positive	Negative	Positive rate ( %)	***P***
CD	30	11	19	36.67	= 0.02	22	8	73.33	= 0.004
Normal colonic tissues	30	20	10	66.67		11	19	36.67	

### Relative expression levels of GPX4 and PTGS2 in CD and normal colonic tissues

**Figure 2:**
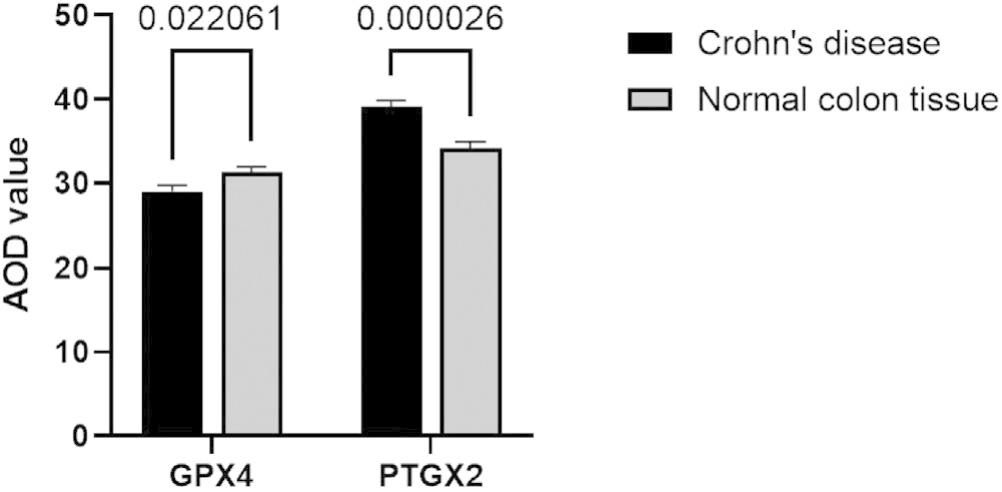
Comparison of the relative expression levels of GPX4 and PTGS2 in the CD and normal colonic tissues.

Based on the average optical density (AOD) values of the positive reactants, the relative expression levels of GPX4 and PTGS2 in CD and normal colonic tissues were analyzed, as shown in Table 3. The relative expression levels of GPX4 and PTGS2 in CD and normal colonic tissue are shown in Figure 2. The relative expression levels of GPX4 in CD colonic tissues were lower than those in normal colonic tissues (*p* = 0.02), while the relative expression levels of PTGS2 in CD colonic tissues were higher than those in normal colonic tissues (*p* = 0.000).

**Table 3:** The expressions of GPX4 and **PTGS2** in the CD and normal colonic tissues.

Group	Case number	GPX4	p	PTGS2	p
CD	30	29.01 ± 7.57	= 0.02	39.05 ± 9.71	= 0.000
Normal colonic tissues	30	31.27 ± 6.78		34.18 ± 9.17

### Correlation between GPX4, PTGS2, and pediatric CD disease activity

The expression levels of GPX4 and PTGS2 in different disease activity stages of CD are shown in Table 4. The staining scores of GPX4 in CD colonic tissues decreased gradually with increasing CD disease activity (*p* < 0.001); the staining scores of PTGS2 in CD colonic tissues increased gradually with increasing CD disease activity (*p* < 0.001). GPX4 staining scores were negatively correlated with pediatric CD disease activity (*r* = −0.326), and PTGS2 staining scores were positively correlated with pediatric CD disease activity (*r* = 0.299).

**Table 4:** The expressions of GPX4 and PTGs2 in different phases of CD.

Case phase	Case number	GPX4	p	PTGS2	p
Mild activity phase	9	31.37 ± 1.32	< 0.001	34.96 ± 1.51	< 0.001
Moderate activity phase	8	30.43 ± 0.90		39.10 ± 1.17	
Severe activity phase	13	25.75 ± 1.15		41.93 ± 1.22	

## Discussions

CD causes serious threats to children's physical and mental health and has become one of the major chronic diseases affecting children and adolescents. Currently, there is no gold standard for the diagnosis of CD. The diagnosis requires a comprehensive analysis combining clinical manifestations, endoscopy, imaging, and histopathology. In this study, the authors collected 30 cases of pediatric CD first diagnosed at the hospital, including 22 males and 8 females, with an average age of 12.36 ± 2.65 years. In the pathogenesis of CD, the integrity of intestinal epithelial cells, tight junctions between cells, and the completeness of the intestinal barrier function are key factors in the onset, progression, and prognosis of the disease [12,13]. In CD affected intestinal segments, vascular damage, reduced oxygen supply, and infiltration of a large number of inflammatory cells, accompanied by a high metabolic state, place intestinal epithelial cells in a hypoxic environment, leading to disrupted intracellular iron metabolism, which induces ferroptosis and activates the body’s inflammatory response, contributing to the inflammatory process [14,15]. Ferroptosis is a newly identified form of regulated cell death caused by the accumulation of iron-dependent ROS and LPO. The gastrointestinal tract is a major source of ROS generated by lipid peroxidation, and due to the low content of antioxidants in the intestinal mucosa, the intestine is more susceptible to oxidative stress-induced ferroptosis [16]. During the process of ferroptosis, GPX4 and PTGS2 are considered biomarkers of ferroptosis and participate in the enzyme-catalyzed lipid peroxidation process. Therefore, investigating the expression of GPX4 and PTGS2 in pediatric CD colonic tissues provides a new perspective for further research into the mechanisms underlying the development and progression of CD.

In this study, immunohistochemistry was used to detect the expression of GPX4 and PTGS2 in colonic tissue by colonoscopy biopsy from 30 pediatric CD cases. The results showed that the positive expression rate of GPX4 in CD colonic tissues was lower than that in normal colonic tissues (*p* = 0.02), while the positive expression rate of PTGS2 in CD colonic tissues was higher than that in normal colonic tissues (*p* = 0.004). The relative expression levels of GPX4 in CD colonic tissues were lower than those in normal colonic tissues (*p* = 0.02), while the relative expression levels of PTGS2 in CD colonic tissues were higher than those in normal colonic tissues (*p* = 0.000). These findings indicate that ferroptosis occurred in the intestinal epithelial cells of CD patients, and that GPX4 and PTGS2 are involved in the ferroptosis process of CD intestinal epithelial cells. During ferroptosis in intestinal epithelial cells, downregulation of GPX4 mRNA and upregulation of PTGS2 mRNA leads to the accumulation of LPO, which causes damage to the cell nucleus and mitochondria, triggering ferroptosis [17]. GPX4 reduces the toxicity of lipid peroxides by converting toxic lipid hydroperoxides into non-toxic lipid alcohols through GSH, maintaining the stability of the cell membrane lipid bilayer and protecting against oxidative damage, thereby inhibiting ferroptosis [18]. PTGS2 is a key rate-limiting enzyme that catalyzes the production of prostaglandins from arachidonic acid. Under normal physiological conditions, PTGS2 is expressed at low levels in most tissue cells, but its expression is increased in response to stimuli such as cytokines and inflammatory mediators, which induce inflammation and promote peroxidase activity, ultimately leading to ferroptosis [19]. Therefore, GPX4 and PTGS2 are key regulators of ferroptosis.

To investigate the correlation between GPX4, PTGS2, and pediatric CD disease activity, this study divided pediatric CD cases into mild, moderate, and severe activity phases based on PCDAI scores. The results showed that the staining scores of GPX4 in CD colonic tissues decreased gradually with increasing CD disease activity (*p* < 0.001), with a correlation coefficient of *r* = −0.326 between GPX4 expression staining scores and disease activity, indicating a negative correlation between GPX4 and pediatric CD disease activity. The progression of CD increases the infiltration of inflammatory cells in the intestine, which induces the production of pro-oxidative molecules, leading to the accumulation of ROS and promoting ferroptosis in intestinal epithelial cells [20]. Studies have shown that IGF2BP2 enhances GPX4 expression through m^6^A modification, inhibiting ferroptosis in intestinal epithelial cells and reducing damage to the colonic tissues of mice [21]. Research by Mayr et al., [22] indicated that, compared with wild-type mice, intestinal epithelial cells with GPX4 deficiency in mice showed reduced GPX4 activity, which induced ferroptosis in the intestinal epithelial cells. A clinical study indicated that selenium supplementation in selenium-deficient populations could enhance GPX4 activity, inhibit ferroptosis in intestinal epithelial cells, and repair intestinal epithelial barrier function, thereby preventing the onset of IBD [23]. The results of this study also showed that the staining scores of PTGS2 in CD colonic tissues increased gradually with increasing CD disease activity (*p* < 0.001), with a correlation coefficient of *r* = 0.299 between PTGS2 staining scores and disease activity, indicating a positive correlation between PTGS2 and pediatric CD disease activity. PTGS2 plays a critical role in lipid peroxidation within cells, promoting ferroptosis [24]. Xu et al., [25] found that increased PTGS2 expression in intestinal epithelial cells led to the accumulation of ROS and LPO, which in turn induced ferroptosis. Traditional Chinese medicine, Astragalus polysaccharide, can reduce PTGS2 expression levels, decrease ROS accumulation and LPO toxicity, inhibit ferroptosis in intestinal epithelial cells, and alleviate intestinal inflammation [26]. Therefore, decreased GPX4 activity and increased PTGS2 expression levels can result in the accumulation of ROS and LPO in cells, inducing ferroptosis in intestinal epithelial cells.

The results of this study showed that the correlation between the expressions of GPX4 and PTGS2 and pediatric CD disease activity was weak. The possible reasons are speculated as follows. For each CD case, only a single lesion tissue from the colonoscopy biopsy specimen was taken from the colonic tissue. This might fail to represent the lesion degree of the entire lesion area, resulting in significant intra-group variability, which could mask the true association strength between the expressions of GPX4 and PTGS2 and pediatric CD disease activity. The staining scores (such as the semi-quantitative method) of immunohistochemical staining rely, to a certain extent, on the observer's subjective interpretation, which may lead to the potential for inter-observer variation. The Pediatric Crohn's Disease Activity Index (PCDAI) score is a comprehensive indicator, including clinical symptoms, laboratory indicators, and colonoscopy findings, etc. Although these indicators have been verified through clinical cases, some components (such as abdominal pain, stool frequency) depend on subjective symptom reporting, especially in children, whose reports may be inaccurate. The magnitude of correlation coefficients is influenced by sample size. A smaller sample size increases random errors, resulting in unstable correlation estimates that may underestimate the true strength of the association.

In conclusion, ferroptosis is a newly discovered form of regulated cell death, characterized by iron-dependent accumulation of ROS and LPO. GPX4 and PTGS2 are considered biomarkers of ferroptosis, participating in the enzyme-catalyzed lipid peroxidation process, which induces ferroptosis. The results of this study show that GPX4 and PTGS2 are involved in the ferroptosis of CD intestinal epithelial cells and are correlated with the severity of CD. These findings provide a new perspective for further research into the role of ferroptosis signaling pathways in the development and progression of CD.

This study focused solely on pediatric CD cases treated at the hospital to investigate the expression of ferroptosis-related proteins GPX4 and PTGS2 in CD and their correlation with CD. However, the sample size was limited, and the source was relatively singular, resulting in certain limitations. Further verification through multi-center clinical studies with larger samples is still necessary. If prospective cohort studies are conducted in the future, with long-term follow-up of pediatric patients, detection of changes in ACSL4 and LPCAT3 at different disease stages in the same patient, and analysis of their correlations with disease recurrence, progression, and treatment, it will be of significant value for the clinical diagnosis and treatment of pediatric CD.

## Financial support and sponsorship

Nil.

## Conflicts of interest

There authors declare no conflicts of interest.
